# Temporal variables improve a spatiotemporal species distribution model for the non-native freshwater fish *Candidia temminckii*

**DOI:** 10.1016/j.isci.2024.109445

**Published:** 2024-03-06

**Authors:** Taichi Jibiki, Shinji Fukuda

**Affiliations:** 1 Graduate School of Agriculture, Tokyo University of Agriculture and Technology, Saiwai-cho 3-5-8, Fuchu, Tokyo 183-8509, Japan; 2 Institute of Global Innovation Research, Tokyo University of Agriculture and Technology, Saiwai-cho 3-5-8, Fuchu, Tokyo 183-8509, Japan; 3 Institute of Agriculture, Tokyo University of Agriculture and Technology, Saiwai-cho 3-5-8, Fuchu, Tokyo 183-8509, Japan; 4 Research Center for Agricultural Information Technology, National Agriculture and Food Research Organization, National Research and Development Agency, Nishi-Shimbashi 2-14-1 Minato-ku, Tokyo 105-0003, Japan

**Keywords:** Environmental science, Nature conservation, Ecology

## Abstract

Ecosystem conservation requires a deeper understanding of species-habitat relationships and population dynamics at a fine spatiotemporal resolution. We propose a new distribution modeling method based on a 5-year monthly survey that considers the temporal continuity of species distributions and physical habitat datasets by inputting continuous time-related variables. We employed random forests to relate the presence/absence of the non-native freshwater fish *Candidia temminckii* to physical habitat data at 15 sampling sites along a 1.4 km spring-fed river in Japan. The proposed method outperforms all conventional methods using datasets split into a specific time period to incorporate temporality into the model. The order of variable importance and shape of the partial dependence plots of the proposed method reflect species ecology and show a gradual shift over time compared to the conventional methods. These results demonstrate the applicability of the proposed method to species distribution modeling using fine-scale spatiotemporal data.

## Highlights

• Data-driven species distribution model considering temporal continuity was built
• The occurrence of non-native *Candidia temminckii* in a spring-fed river was modeled
• The proposed model outperforms the models using dataset in a specific time period
• Species ecology extracted from the proposed model shows a gradual shift over time

## Graphical abstract

**Figure:**
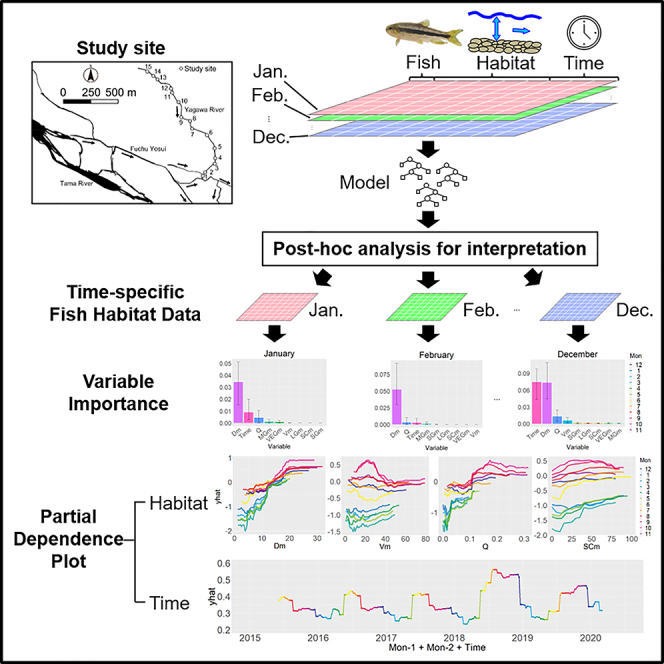

## Introduction

Freshwater ecosystems are expected to face greater challenges than terrestrial and marine ecosystems (e.g., Tickner et al.^1^). There is a need to understand the information required for freshwater ecosystem conservation^2^^,^^3^ and develop methods for data acquisition and analysis at detailed spatiotemporal scales. Fish populations that migrate and disperse across continuous waters are more susceptible to the structures of their habitats and river networks than terrestrial species that can move over land.^4^ Therefore, it is important to collect and compile data on river and stream networks and connectivity as well as the distribution of crossing structures^5^ as a mobility barrier for freshwater ecosystems (see Grill et al.^6^ for instance of free-flowing rivers). In conjunction with the structures, distributions, and connectivity of riverine environments, understanding population dynamics, such as migration, prompted by external environmental factors of fish, is needed.^7^ In this context, species distribution modeling can contribute to the understanding of population dynamics,^8^ and there is a need to investigate and analyze riverine ecosystem dynamics at detailed spatiotemporal scales, which have been challenging during the last few decades.^4^^,^^9^

Temporal information is essential for modeling species distributions^10^ because species distributions, in general, are influenced by various factors that fluctuate over space and time.^11^ As a result, methods that include temporal information in species distribution modeling are being developed.^12^ Phenology is a periodic biological phenomenon associated with seasonal environmental changes and is an important concept when considering species distribution.^13^ However, the effects of phenology have often been neglected or ignored in species distribution modeling.^14^ Therefore, it is important to develop a modeling method that considers species phenology. The consideration of temporal information becomes more prominent when modeling species distributions for a longer period, such as a year or more (see Ryo et al.^15^ for an instance). The impact of time on the results of species distribution modeling was addressed in the context of temporal transferability. The distribution pattern of a species changes over time owing to changes in its population dynamics and dispersal.^16^

While species distribution modeling that considers temporal information on a detailed spatiotemporal scale is important for understanding the dynamics of fish populations in rivers and streams, no long-term study has implemented it using detailed spatiotemporal field data. In many cases, species distribution modeling at broad spatial scales over multiple years does not consider temporal information (e.g., McNyset^17^; Huang et al.^16^). Furthermore, while some methods consider phenology in species distribution modeling, there is room for improvement. Traditionally, phenology has been considered in species distributional modeling by partitioning a dataset by the temporal scale of interest and building a set of models for each data subset (e.g., Gschweng et al.^18^; Baltensperger and Joly^19^). We define this method as the conventional method in this article. However, because phenology is a phenomenon caused by temporally continuous changes in individual body conditions and environmental gradients,^14^ splitting the data makes it difficult to take into account the temporal continuity of the data when interpreting the results from the model. It is also necessary for the modeler to determine the temporal interval to split the data before constructing a species distribution model. When modeling ecological data with temporal continuity, temporal information should be input as a continuous variable.^20^ The use of temporal information as a model input allows the consideration of species phenology without losing the temporal continuity of the dataset used; however, it is necessary to use a model that can account for variable interactions. Random forests (RF^21^), which is one of the commonly used methods in species distribution modeling, accounts for higher-order interactions among multiple variables in geospatial-scale species distribution modeling.^22^ RF also showed a higher predictive performance than the other models in detailed spatial-scale modeling.^23^

Therefore, we propose a novel approach to species distribution modeling that incorporates species phenology and its temporal continuity for better predictive modeling by inputting continuous time-related variables. We define this method as the proposed method in this article. In this way, we aim to enhance understanding of species distributions and movement patterns in response to specific instream habitat conditions. Previous studies have considered phenology by inputting variables representing seasons, but they do not consider temporal distances for different months or years (Williams et al.^24^). The temporal distances can be exemplified as follows. For example, the temporal distance between May and July should be greater than that between May and June (monthly scale), and the temporal distance between 2017 and 2019 should be greater than that between 2017 and 2018 (annual scale). Whereas the habitat range of the target species and spatial scale of the data used may be different, the consideration of temporal distances in species distribution modeling allows for better interpretation of time-related information in species-habitat relationships and their dynamics than previous studies.

**Figure 1:**
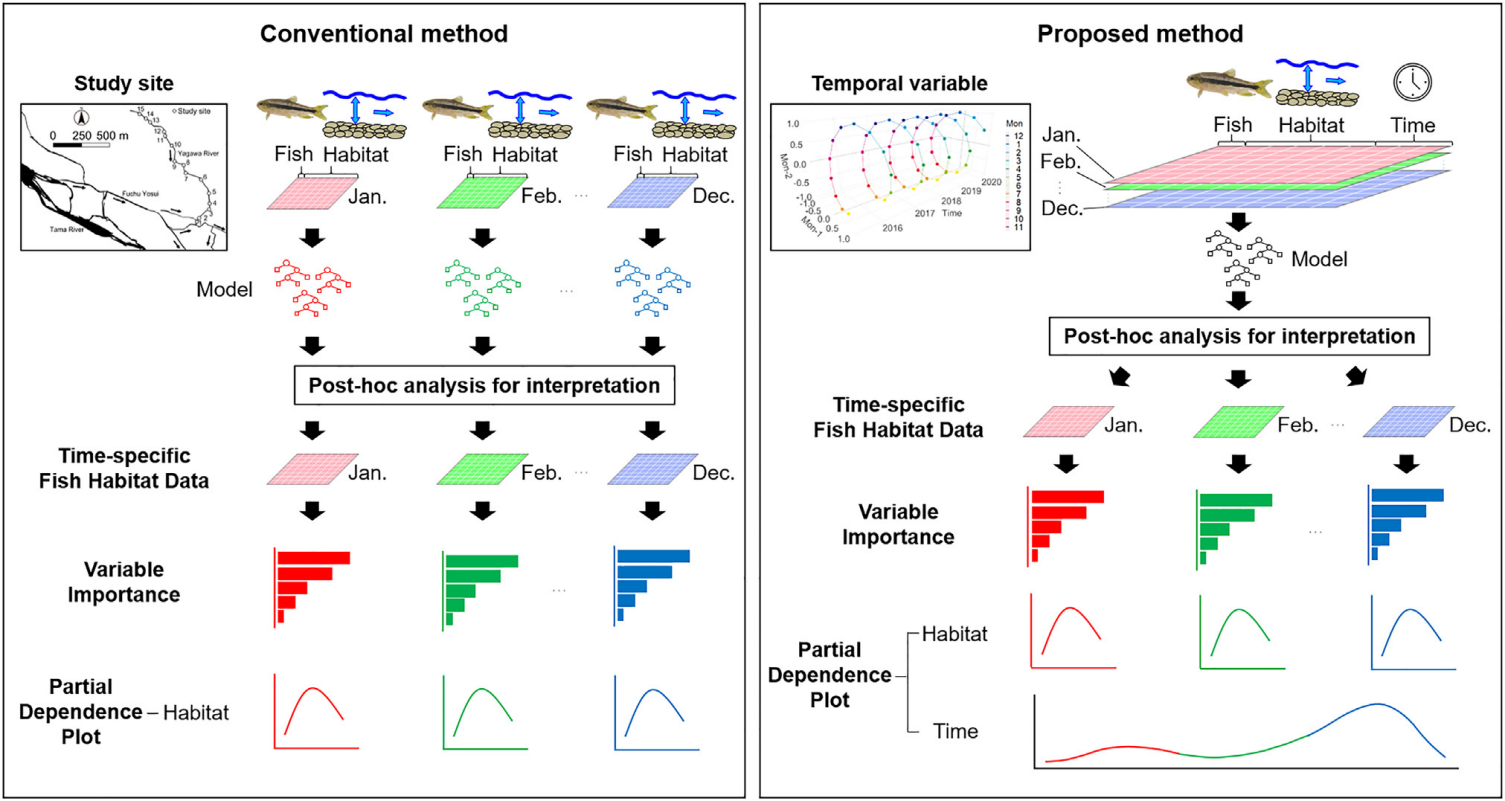
Conceptual diagrams displaying the conventional and proposed methods of *post hoc* analysis Conventional methods consider temporal information by dividing the data into time periods of interest and constructing models for each period. The proposed method considers temporal information by inputting a variable that indicates temporal distances. In the conventional species distribution model, variable importance and partial dependence plot for a specific period were assessed by applying the model built using the training data to the *post hoc* test data. In the proposed method, variable importance and partial dependence plot for a specific period were computed by applying the model built using the entire training dataset to the *post hoc* test data in a specific period. Compared to conventional methods, the proposed method does not lose the temporal continuity of the distribution data of the target species and physical habitat data; thus, it is possible to discuss the results based on their temporal continuity. Since the proposed method inputs time-related variables as continuous variables, it is possible to calculate variable importance and partial dependence plot for the time-related variables.

In this study, we applied machine learning to analyze spatiotemporally detailed species distributions and physical habitat data to understand the factors affecting the distribution and population dynamics of a target fish species (Figure 1). The key questions in this study are 3-fold: (1) whether the proposed method is superior to conventional methods in terms of accuracy and interpretability; (2) what kind of population information can be obtained from the proposed method using spatiotemporally detailed species distribution and instream physical habitat data; and (3) how time-related variables work in the proposed method. Specifically, a model for predicting the presence or absence of fish was constructed using RF based on the instream physical habitat conditions collected monthly from 2015 to 2020 at 15 survey sites along a small spring-fed river in Tokyo, Japan. The model performance was evaluated for four cases of the conventional species distribution modeling method with varying data partition widths and for one case of the proposed method, and the model performance of each method was compared. *Post hoc* analyses were also conducted for the conventional and proposed species distribution modeling methods to compare the results of each method and discuss the population dynamics of dark chubs in the river. We also discuss the possibility that the proposed method contributes to the study of basic parameters of population dynamics, such as reproductive and mortality rates.

## Results

### Prevalence and physical habitat conditions

Results of fish sampling and physical habitat surveys conducted in the target river are as follows. Both surveys were conducted monthly in the study area between June 2015 and March 2020. Fifteen sampling sites, 10-m long, were set up in the target river (Figure S1). The target river is a spring-fed river, approximately 1.4 km long, which flows through Kunitachi, Tokyo, Japan. The target fish species was the dark chub (*Candidia temminckii*). The spawning season of the target fish was from May to August, and sand and gravel were used as the spawning beds.^25^

**Figure 2:**
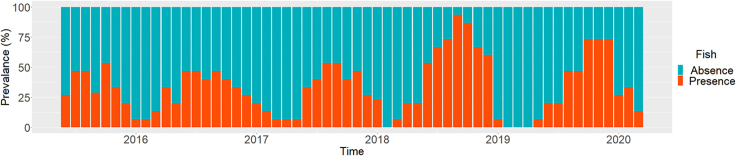
Change in data prevalence (i.e., the percentage of sites where the target fish *Candidia temminckii* were caught) per survey

Seasonal trends and annual fluctuations influenced the prevalence (i.e., the percentage of sites where fish were caught) of the dark chub changed over time (Figure 2). We observed a seasonal tendency of low prevalence from January to May and high prevalence from June to December. The prevalence fluctuated over the years, even within the same month. For instance, the prevalence increased in 2018 and 2019 following the flow intermittency in February.

**Figure 3:**
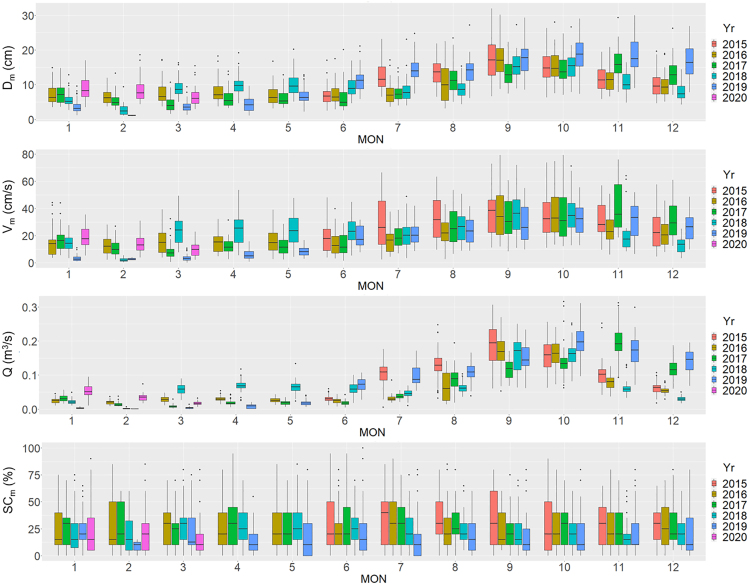
Temporal dynamics of the physical habitat conditions: mean depth (*D*_m_), mean velocity (*V*_m_), flow discharge (*Q*), and percent coverage of silt and sand (*SC*_m_) The horizontal axis of the figure indicates the month (MON) in which the data were acquired.

Physical habitat characteristics, such as water depth, water velocity, and flow discharge, exhibit seasonal and annual dynamics (Figure 3). We observed a minimum flow in January and February, an increasing flow from June to August, a maximum flow between September and November, and then a decreasing flow from November to December. There were differences over the years, but monthly fluctuations were more prominent. The flow regime of the Yagawa River depended on the rainfall in the catchment, indicating diverse hydrological fluctuations over time. Variations in the substrates were mainly ascribed to the spatial distribution of different materials along the river, which remained similar throughout the year (Figure S3).

We observed a temporal change in prevalence without distinct changes in physical habitats. For instance, scatterplots between water depth and percent coverage of silt and sand for two consecutive months, May and June 2018, showed that the physical habitat conditions were nearly the same, but the data prevalence increased (Figure S4). A similar trend was found for water depth and velocity for the same month in October 2016 and October 2018 (Figure S5).

### Species distribution models

We built species distribution models using RF,^21^ a machine learning method, to model and interpret the distribution patterns of dark chubs in the Yagawa River. Here, we compared the accuracy and *post hoc* analysis results of the conventional species distribution modeling method and the proposed method. Conventional methods consider temporal information by dividing data into time periods and constructing models for each period. The proposed method considers temporal information by inputting a variable that indicates it. The response variable of the models was the presence/absence of the dark chub in each sampling site, while explanatory variables are various physical habitat variables (mean depth, mean velocity, flow discharge, percent coverage of aquatic vegetation, large-sized gravels, medium-sized gravels, small-sized gravels, and silt and sand). In addition to the physical habitat variables, time-related variables, namely time (Time) and month (Mon-1 and Mon-2), were introduced in the proposed species distribution models to consider temporal dynamics in species-habitat relationships. The incorporation of the time-related variables into species distribution models is the most novel aspect of this study. The monthly variable describes periodic similarities. To represent a cycle, two variables (Mon-1 [x axis] and Mon-2 [y axis]) were used and positioned equally spaced clockwise from the starting point ([x, y] = [0, 1]) in a circle with a radius of 1 (Figure S2A). The time variable (Time) indicates the temporal continuity (Figure S2B), defined as

Time = Year + (Month–1)/12.

The accuracy of each model was evaluated using multiple performance measures (namely, area under the receiver operating characteristic [ROC] curve [AUC], correctly classified instances [CCI], mean squared error [MSE], and Nash-Sutcliffe efficiency [NSE]). To interpret the models, *post hoc* analyses (i.e., variable importance and partial dependence plots) were conducted.

**Figure 4:**
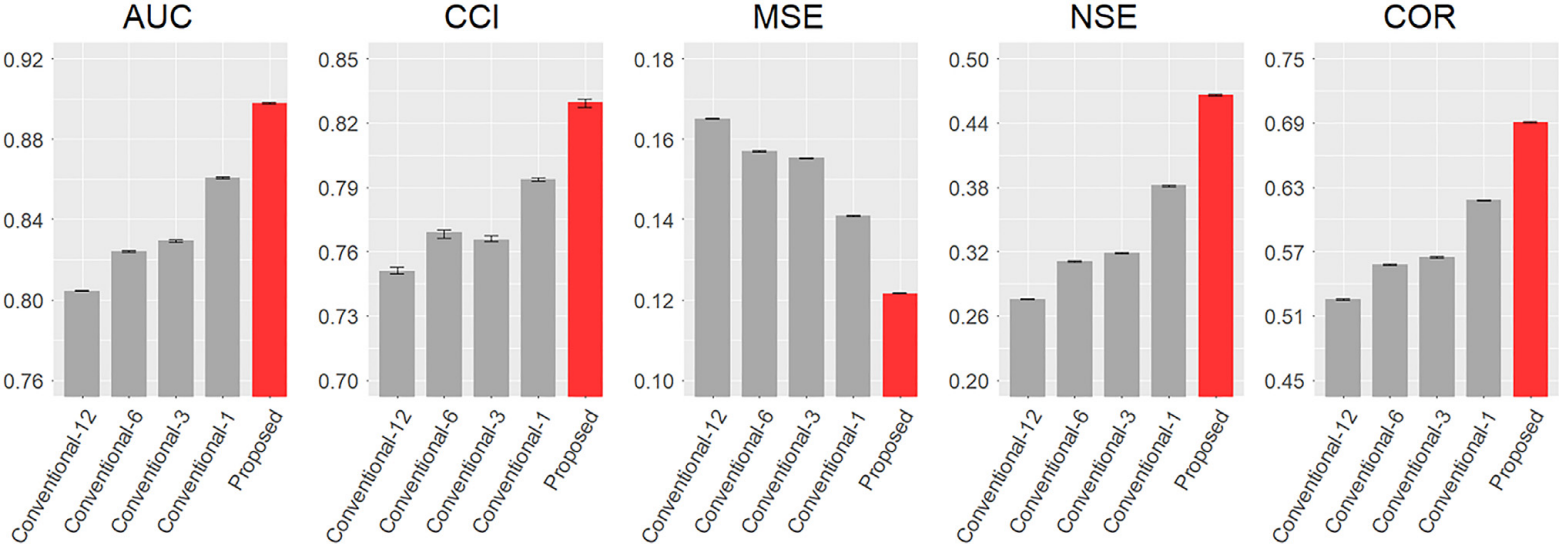
Model performance of species distribution models with respect to multiple performance measures, namely AUC (area under the ROC curve), CCI (correctly classified instances), MSE (mean squared error), NSE (Nash-Sutcliffe efficiency), and COR (correlation coefficient) The values of the bars represent the mean performance calculated based on the results of 10-fold cross-validation. The error bars indicate the maximum and minimum values of each performance measures.

After calibration, we obtained the optimal hyperparameters of RF, namely, mtry = 3 and ntree = 5,000, and used them in the following analyses. The proposed method outperformed the conventional methods in accuracy (Figure 4). For the conventional species distribution modeling method, four cases with different monthly split widths were set up to evaluate the impact of the time width over which the data were split on the accuracy of the model (Table S1). The numbers given to the names of the conventional method cases indicate the number of months included in each split of data. For example, Conventional-1 is modeled by dividing the data by one month. The model performance of the conventional methods was better for the method with a shorter time window, namely Conventional-1.

*Post hoc* analysis was performed for each of the proposed and conventional methods, and the characteristics of each method were compared based on those results. For the *post hoc* analysis, variable importance and partial dependence plot were calculated.

**Figure 5:**
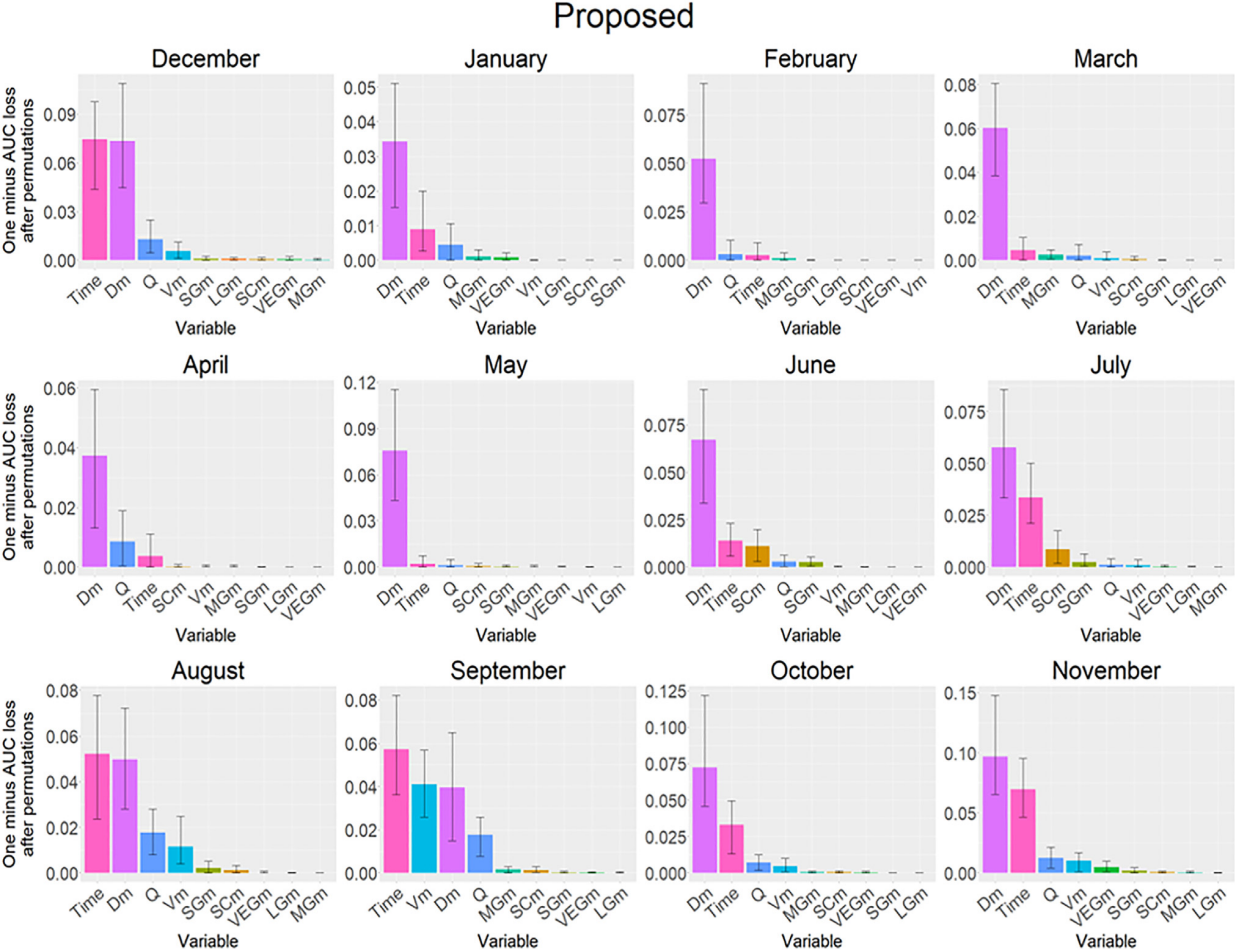
Variable importance by month using the proposed method The y axis shows the importance of each variable in the model prediction. The bars represent the mean variable importance calculated by 100 times permutation, and the error bars indicate the maximum and minimum values of importance. Explanatory variables are mean depth (*D*_m_), mean velocity (*V*_m_), flow discharge (*Q*), percent coverage of aquatic vegetation (VEGm), large-sized gravels (LGm), medium-sized gravels (MG_m_), small-sized gravels (SG_m_), and silt and sand (SCm).

Variable importance is the contribution of each variable to the model prediction. The variable importance of the conventional and proposed methods varied with time, and the water depth was found to be important throughout the year (Figures 5, S6, and S7). The time variable introduced in the proposed method remains important throughout the year. The major difference between the conventional and proposed methods is the magnitude of change in the relative importance over time, with the conventional methods showing a greater shift than the proposed method. Whereas Conventional-3 cannot compute variable importance at a finer timescale as the Conventional-1, temporal shifts in variable importance are smoother for Conventional-3 than for Conventional-1.

**Figure 6:**
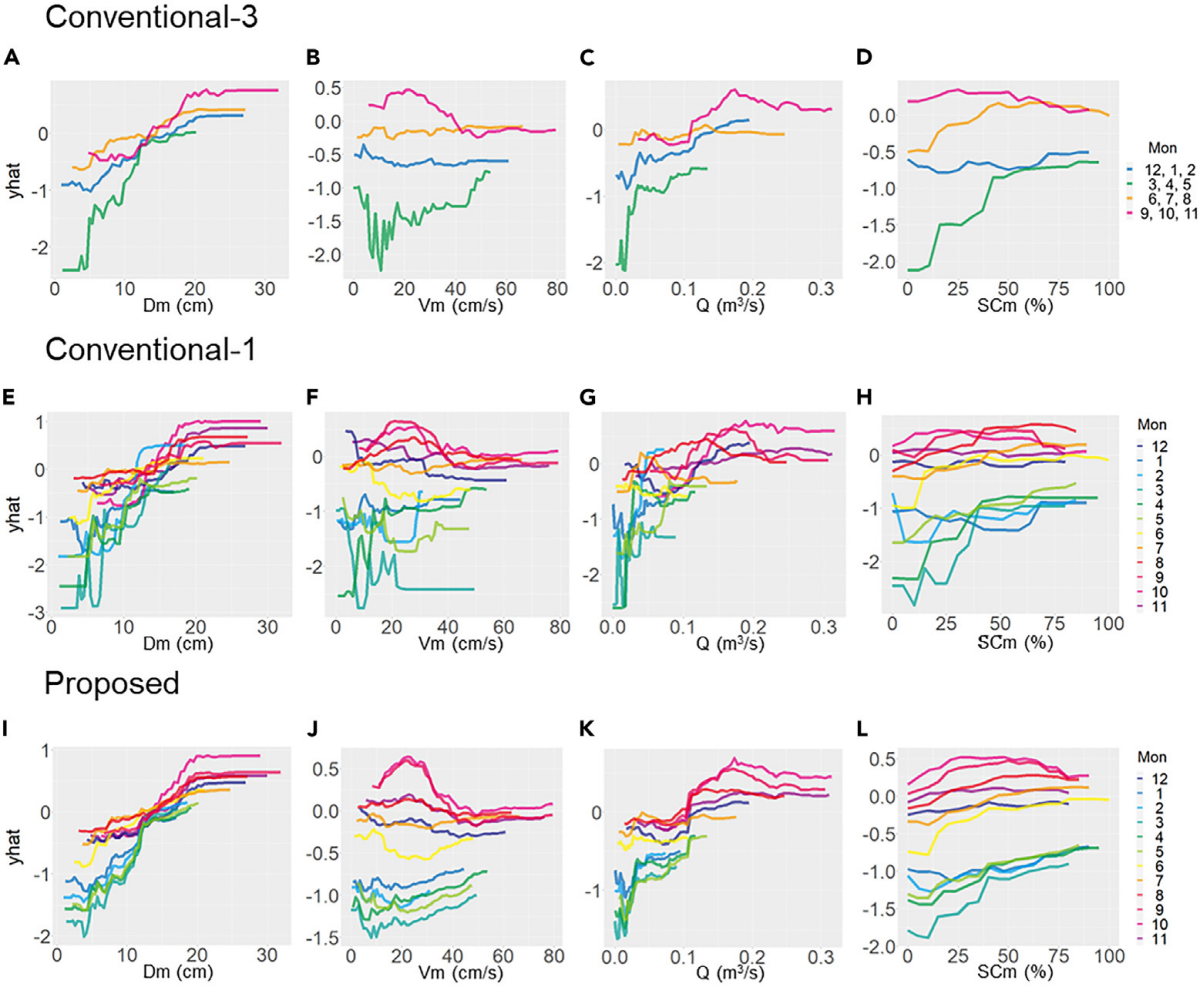
Monthly partial dependence plots for key variables, namely depth (*D*_m_; A, E, and I), velocity (*V*_m_; B, F, and J), discharge (*Q*; C, G, and K), and percent coverage of sand and clay (SC_m_; D, H, and L) by conventional (Conventional-3 and Conventional-1) and proposed methods, respectively Partial dependence plots show the relationship between the model predictions with changes in the values of the explanatory variables. The vertical axis shows the trend of change in the model prediction.

**Figure 7:**
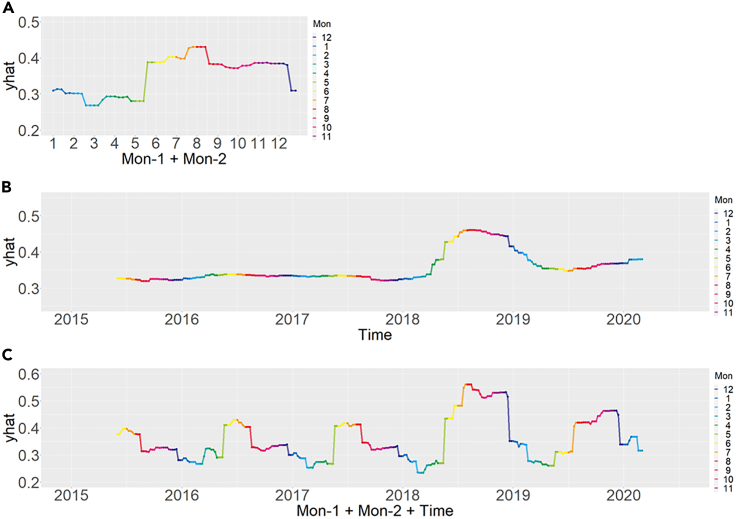
Partial dependence plot for time-related variables (A) Month, (B) Time, and (C) Month + Time. Only the proposed method can calculate partial dependence plot for time-related variables. Monthly cycle is described by a bivariate function using Mon-1 and Mon-2 (Figure S2).

Partial dependence plots show the relationship between the model’s predictions with changes in the values of the explanatory variables. The shapes and magnitudes of the partial dependence plots changed with time for both the conventional and proposed methods (Figures 6 and S8). While the proposed method exhibits partial dependence plots with smoother shapes and shifts in magnitude, the partial dependence plots obtained by conventional methods are complex, variable, and inconsistent. As in the case of variable importance, the temporal shifts in the partial dependence plots were smoother for Conventional-3 than for Conventional-1. Only the proposed method can calculate partial dependence plot for time-related variables. The partial dependence plot for the monthly variables (i.e., Mon-1 and Mon-2) indicated a low likelihood from January to May and a large increase from June to December (Figure 7A). The partial dependence plot for time indicated a low likelihood in 2015–2017, an increase in 2018, a decrease in 2019, and a slight increase in early 2020 (Figure 7B). The composite partial dependence plot for the time and month variables appeared to reflect the temporal fluctuation of data prevalence (Figure 7C).

## Discussion

### Importance of temporal information

Given the importance of temporal information in species distribution models, there is a need to investigate and analyze species distributions on a fine spatiotemporal scale. Moreover, conventional methods that consider phenology by splitting data according to the time of interest cannot satisfy temporal continuity of the distribution data of target species and physical habitat data. In this study, we introduced time-related variables (Month and Time) into a species distribution model using RF, which considers variable interactions. The proposed method outperformed conventional methods, and the results of *post hoc* analyses showed that the distribution patterns over time were well represented in the proposed method. For instance, the proposed method captured changes in conditions, such as individual sexual maturity and total length of individuals in the Yagawa River, and the distribution of the target species was well explained by the instream habitat conditions, namely water depth.

The temporal variables contributed to the *post hoc* analyses, and the variable importance and partial dependence plots obtained by the proposed method were smoother and consistent over time. The results also suggest that the output of the proposed method indirectly represents the population dynamics of the target species and can be used as an indicator of the reproductive rate and mortality, which are the basic parameters of population dynamics. Further studies are needed to test the use of the outputs of species distribution models as explanatory variables for modeling the population dynamics of a target species.

### Model performance

The proposed method, in which temporal information is considered, outperformed conventional methods and alleviated the overfitting problem partly observed in the conventional methods. The proposed method, in which one model was built with all data by considering time-related variables, is more accurate than the conventional methods, which divide data by time period and create a model for each divided data. This is consistent with previous research (Williams et al.^24^) which showed that a model created with all data with seasonal information input as a variable is more accurate than a model created by splitting data by season. Among the conventional methods, the model performance indicated that the accuracy improved for shorter time windows (Figure 4). This was primarily because the mixture of varying distribution patterns over time was reduced by splitting the data into specific periods. It has been reported that the habitat preferred by fish species changes seasonally (Carter et al.^26^; Nagayama et al.^27^). Therefore, if data from a wide range of seasons are used as training data for the same model without considering seasonal information, a wide range of distribution patterns will be mixed in the training data. In addition, dataset with a larger sample size improves the accuracy of the model (Hernandez et al.^28^; Thibaud et al.^29^). However, in the case of the dataset used in this study, the mixed patterns of species distributions within the data for model training are considered having a greater impact on model performance than the sample size per model. When using the conventional methods, the time window for splitting data should be appropriately adjusted to balance the model performance and interpretability of the *post hoc* analyses results.^19^ In contrast, the proposed method can model temporal dynamics using temporal variables (i.e., Month and Time), in which month reflects a monthly cycle of species response in a year and time reflects the annual fluctuation of species distributions in the target river. The contribution of the time-related variables to species distribution modeling is described in detail later.

### Transferability of ecological information

The results of the *post hoc* analysis (variable importance and partial dependence plots) computed by the conventional and proposed methods suggest a trade-off between accuracy and generalization ability (i.e., transferability) in species distribution modeling. The proposed method is considered a promising approach to overcome the trade-off.

Comparing the variable importance between the conventional and proposed methods (Figures 5, S6, and S7), the variable importance of the conventional methods for a single month (Conventional-1) was inconsistent over time rather than the proposed method. For instance, in the conventional methods, the importance of flow discharge in April, as well as the percent coverage of silt and sand in June, was significantly high, but only in those respective months. The variable importance computed using the proposed method was mostly consistent over time, in which the water depth, velocity, discharge, and percent coverage of sand and clay were the major variables. Regarding the partial dependence plots, the shapes obtained by the conventional methods were variable and inconsistent compared to those of the proposed method, specifically in winter (Figures 6 and S8).

In contrast, the partial dependence plots obtained using the proposed method had smooth shapes and were consistent over time. These results support the validity of the proposed method from an ecological viewpoint, with a specific consideration of temporally smooth shifts in the species-habitat relationship. Regarding the partial dependence plots in the conventional methods, the sharp variance rather than smooth shape of a partial dependence plot for a particular month is also unnatural, given the general pattern of species distributions. The inconsistency in the results of the *post hoc* analysis in the conventional methods may be due to the reduction in the number of sample sizes per model due to data splitting by season. The reason why model performance generally declines with sample size could be that the level of uncertainty associated with parameter estimates increases as sample size decreases (Wisz et al.^30^). Specifically, outliers can have a stronger influence on the analysis, but a dataset with a larger sample size reduces such influences by buffering the effects of outliers. Since the conventional methods split data by time period, the proportion of outliers per data group can be greater, which makes the results of the *post hoc* analysis difficult to interpret. In the conventional methods, interpretation is particularly difficult in winter when the prevalence is low. However, when comparing the accuracy between cases of conventional methods with different time windows, the accuracy tended to be higher in cases with smaller time windows. This implies that conventional methods tend to capture species-habitat relationships specific to a particular time period (including noise), and thus the models may lose generalization ability as a result of overfitting. This is a trade-off between the accuracy and generalization ability of the conventional methods. In contrast, the proposed method is more consistent over time as shown in the results of the *post hoc* analysis, and its model performance is higher than that of the conventional methods. Therefore, the proposed method is considered a promising approach to overcome the trade-off between the accuracy and generalization ability of species distribution modeling.

### Habitat characteristics of the dark chub

The results of the *post hoc* analyses of the proposed method are noteworthy because the importance of percent coverage of silt and sand in June and July increases. The partial dependence plot shows that the likelihood of dark chub presence increased with increasing silt and sand coverage during this period (Figure 6). This is supported by the fact that the dark chub spawning season overlaps with this period, and its spawning habitat can be sand or gravel.^25^ Nagayama et al.^27^ reported the importance of sandy substrates for spawning dark chubs in June. The reproductive condition, which is an individual eco-physiological condition, was evident in the results of the *post hoc* analysis, indicating the potential application of detailed spatiotemporal-scale species distribution modeling to elucidate the ecological traits of the target species.

Next, the importance of water velocity increased from July to September (Figure 5). The partial dependence plot for this period showed that habitat quality was high at relatively slower water velocities (less than approximately 20 cm/s) (Figure 6B). This may be due to a sudden change in the total length of the dark chub population after the emergence of young individuals. In general, smaller individuals have limited swimming ability and, therefore, prefer lower water velocities.

Species distribution modeling on a fine spatiotemporal scale may partially reflect population dynamics (changes in total length) based on the presence/absence data of the target species. The most important variable was water depth, and deeper water was more suitable for this species in the Yagawa River. Because the Yagawa River is small and there are many sampling sites with shallow water depths (<20 cm), the importance of water depth may be dominant throughout the year.

As described earlier, the proposed method, which explicitly incorporates temporal continuity in species distribution modeling, allows for capturing the changes in the ecological and physiological conditions of a fish population. This information may be useful for modeling the distribution and migration of dark chubs.

### Habitat prediction and population dynamics

Monthly cycles explain the seasonal periodicity of species distribution, which is the key to model interpretation from an ecological viewpoint. Because the monthly cycle was expressed as a continuous bivariate function (Mon-1 and Mon-2), the results of the *post hoc* analysis were consistent over time. Temporal information was introduced to represent population dynamics over the observed years, which allowed for consideration of information that could not be explained solely by environmental variables (e.g., changes in the total length of a population or changes in density).

Because the body conditions of dark chub individuals in the Yagawa River (e.g., total length, body weight, and sexual maturity) change with time in accordance with their life stages, temporal information is required for accurate and reliable predictions. The temporal changes in body conditions may be described by the interaction between the monthly cycles (i.e., Mon-1 and Mon-2) and other physical habitat variables, which is supported by similar changes in the shape of the partial dependence plots in consecutive months (Figure 6).

In the case of a small river such as the Yagawa River, a larger population density can result in a wider distribution, even in environments with relatively low habitat quality. For example, the scatterplots comparing October 2016 and October 2018 showed that the instream habitat conditions such as depth and velocity were similar, but the prevalence greatly increased in 2018 (Figure S5). This may be ascribed to the larger population size in October 2018, which resulted in a wider range of species distributions than those in October 2016. The expansion of a species’ range associated with an increased population density is an important factor in defining the spatial distribution of a species.^31^ The partial dependence plot for the variable indicating time (Time) implies temporal changes in the threshold converting the model output (i.e., likelihood of species occurrence) into the presence/absence of a species, supporting Huang et al.^16^ in which the threshold of a species distribution model needs to be well calibrated to predict shifts in species distribution over time.

The importance of the time variable was greater in summer, particularly from June to August. This may imply that the dynamics of instream habitat conditions remained for consecutive years and were insufficient to fully explain the shift in species distribution, which could be partly due to successful reproduction or re-establishment of the population in 2018 after flow intermittency in early 2018. Winter was less important because the instream habitat conditions within the Yagawa River were below the minimum habitat requirement for the dark chub, resulting in a smaller population and narrower distribution of the dark chub. As such, the time variable has the potential to better explain species distribution and population dynamics.

### Conclusions

This study proposes a new method using *post hoc* analyses for data-driven species distribution modeling based on fine-scale spatiotemporal species distribution data. The results supported the applicability of modeling species distributions over time and interpreting species ecology, such as ecological information and community composition of the dark chub population, from presence/absence data in the Yagawa River. The relationship between habitat conditions and population dynamics in the irrigation canal network connected at the downstream end is beyond the scope of this study. However, it is important to comprehensively investigate and elucidate the species-habitat relationship and migration between the river and channel network. For instance, changes in the partial dependence plot for the Month between May and June may be related to the beginning of irrigation in the Fuchu Yosui irrigation system, but this cannot be fully interpreted based on the data of this study alone.

By marginalizing the direct effects of Month and Time variables on the predictions of the species distribution model, it may be possible to evaluate the effects of instream habitat conditions on ecological status such as spawning and mortality of dark chubs at a given location and time period. This study demonstrated that the Month and Time variables could partly explain the population dynamics at a given site, implying that the Month and Time variables provided complementary information to instream habitat conditions. Thus, it may be possible to separate species distribution data into two parts: physical habitat conditions and temporal fluctuations, which can be used for modeling population dynamics and provide fundamental information for the management and control of a species or community. Further studies are needed to relate habitat potential or habitat suitability assessed using a species distribution model to the ecological status of an individual or population of a target species (see Champion et al.^32^ for an instance), based on which the applicability of species distribution models can be further examined.

### Limitations of the study

In this study, habitat quality outside the study area was not considered, which may increase the uncertainty in predicting species distribution within the study area. The distribution potential of a target species at a given location is determined by a gradient of habitat quality within the home range of an individual. Fish individuals are mobile and thereby keep migrating in and out of the study area to find a better habitat for fulfilling their life cycle. Therefore, even if the habitat quality is suitable and constant in the study area, the distribution potential therein can vary depending on the habitat quality around the study area. Further study considering the habitat quality in the Fuchu Yosui irrigation system may illustrate how gradients of habitat quality in and around the Yagawa River can affect the species occurrence.

In addition, we could not consider all the variables that have significant impact on species occurrence. For example, water temperature was not considered though it has a significant impact on the habitat potential of a species. As the Yagawa River is a spring-fed stream, water temperature dynamics are different from the Fuchu Yosui irrigation system which extracts water from the Tamagawa River. In summer, water temperature in the Yagawa River is lower than that of the Fuchu Yosui irrigation system, whereas the trend becomes opposite in winter.

The age structure of a fish population (e.g., composition of total lengths) and its temporal changes are important when interpreting the effects of physical habitat variables on population dynamics. However, population structure such as that represented by total length was not incorporated as an input variable in this study. Here, we proposed the use of temporal variables for representing the effects that change over time. The month variable considers monthly cycle of a temporal component, which can be used for forecasting. However, the time variable is one-dimensional, continuous variable representing both years and months, which cannot be used for forecasting. When forecasting the future, one possible method is to use the average of the predicted values for a month from the time variables for the same month over the past years.

## STAR★Methods

### Key resources table

**Table:** 

REAGENT or RESOURCE	SOURCE	IDENTIFIER
**Software and algorithms**		

R (Version4.0.3)	R Core Team^37^	https://www.r-project.org/index.html
Rstudio (Version2022.7.0.548)	RStudio Team	https://support--rstudio-com.netlify.app/
randomForest (Version4.6.14)	Liaw and Wiener^36^	https://cran.r-project.org/web/packages/randomForest/randomForest.pdf
Splitstackshape (stratified cross validation) (Version1.4.8)	Mahto^38^	https://cran.r-project.org/web/packages/splitstackshape/splitstackshape.pdf.
DALEX (variable importance) (Version2.0.1)	Biecek^39^	https://jmlr.org/papers/v19/18-416.html
pdp (partial dependence plot) (Version0.7.0)	Greenwell^40^	https://journal.r-project.org/archive/2017/RJ-2017-016/index.html

### Resource availability

#### Lead contact

Further information and requests for resources and reagents should be directed to and will be fulfilled by the lead contact, Shinji Fukuda (shinji-f@cc.tuat.ac.jp).

#### Materials availability

This study did not generate new unique reagents or materials.

#### Data and code availability

• Data reported in this paper will be shared by the lead contact upon request.
• Code reported in this paper will be shared by the lead contact upon request.
• Any additional information required to reanalyze the data reported in this paper is available from the lead contact upon request.

### Method details

#### Survey area and target fish

The target site was Yagawa River, which flows through Kunitachi, Tokyo, Japan (Figure S1). The Yagawa River is a spring-fed river, approximately 1.4 km in length, and its downstream end is connected to the Fuchu Yosui irrigation system. The Fuchu Yosui irrigation system is an agricultural canal that receives water from the Tama River and rejoins it, where water from the Tama River flows downstream for irrigation from late May and late September. The Fuchu Yosui irrigation system has permanent water areas where water from the Yagawa River flows even during the non-irrigation period, and temporary water areas where water taken from the Tama River flows only during the irrigation period. No structures within the study area interfered with fish migration.

The target fish species was the dark chub (*Candidia temminckii*). The spawning season is from May to August, and sand and gravel as spawning beds.^25^ Its distribution range is expanding as a domestic invasive species in Japan,^33^ and the populations distributed in and around the Yagawa River are also non-native populations. Dark chubs are one of the dominant species in the Yagawa River and use the Fuchu Yosui irrigation system as a habitat.^34^

#### Fish habitat survey

Fifteen sampling sites 10-m in length were set up in the target river (Figure S1). Fish sampling and physical habitat surveys were conducted monthly in the study area between June 2015 and March 2020.

In the fish sampling survey, fyke nets were set at both boundaries of the sampling sites to prohibit the movement of fish in and out of the sampling sites, and hand nets were used to capture the fish for 10 min. The captured fish were identified as species and released alive.

For the physical habitat survey, cross-sections were set up every 5-m within each 10-m sampling site, and channel width (cm), percent coverage of aquatic vegetation (%), and substrate (%) were recorded. Substrates were classified as large gravel (diameter> 64 mm), medium gravels (16–64 mm), small gravels (2–16 mm), and silt and sand (<2 mm), and the percent coverage of each on the cross-sections was visually recorded. The percent coverage of aquatic vegetation was also visually recorded as a percentage of the cross-section. Water depth (cm) and velocity (cm/s) were measured at five equally spaced points on the cross-section. Water velocity was measured using an electromagnetic current meter (LP30, KENEK, Japan) and averaged five times over 5 s at each measurement point. The water velocity at each measurement point is the average of three measurements, excluding the maximum and minimum values of the five measurements. The flow volume (m^3^/s) at each cross-section was calculated.

#### Species distribution model

Random forests^21^ (RF), a machine learning method, was used to model and interpret the distribution patterns of dark chubs in the Yagawa River. Here, we compared the accuracy and post-hoc analysis results of the conventional species distribution modeling method and the proposed method. Conventional methods consider temporal information by dividing data into time periods and constructing models for each period. The proposed method considers temporal information by inputting a variable that indicates it.

Random forests is an ensemble learning method that uses multiple classification and regression trees. This model is characterized by high computational speed, resistance to outliers, and the ability to analyze correlated but ecologically important variables.^35^ An important feature of the model used in this study is its ability to consider higher-order interactions between variables without prior specification.^22^ To consider temporal information with the input variables in the proposed method, it is necessary to use a model that considers the interactions between variables. The model also showed higher accuracy than the other models in modeling species distributions on detailed spatial scales.^23^ Therefore, random forests were selected from the many existing species distribution models. We used the “randomForest” package^36^ of the R software.^37^

#### Conventional and proposed methods for considering temporal information

Multiple cases were considered to compare the proposed and conventional methods (Table S1). In the conventional method, a model was constructed for each split month, and post-hoc analyses were performed for each model to interpret the distribution pattern of the split months (Figure 1). First, for the conventional species distribution modeling method, four cases with different monthly split widths were set up to evaluate the impact of the time width over which the data were split on the accuracy of the model (Table S1). Next, for the proposed method, one case was considered. The proposed method builds one model with all the data and interprets the pattern of each period in post-hoc analyses (Figure 1).

#### Response and explanatory variables

The response variable was the presence/absence of the dark chub in each sampling site, while explanatory variables are mean depth (Dm; cm), mean velocity (Vm; cm/s), flow discharge (Q; m^3^/s), percent coverage of aquatic vegetation (VEGm; %), large-sized gravels (LGm; %), medium-sized gravels (MGm; %), small-sized gravels (SGm; %), and silt and sand (SCm; %). The mean depth and velocity are the averages of the depth and velocity at the measured cross-section, respectively. In addition to the physical habitat variables, temporal variables, namely (time) and month (Mon-1 and Mon-2), were introduced into the proposed species distribution modeling method. The monthly variable describes periodic similarities. To represent a cycle, two variables (Mon-1 (x axis) and Mon-2 (y axis)) were used and positioned equally spaced clockwise from the starting point ([x, y] = [0, 1]) in a circle with a radius of 1 (Figure S2A). Mon-1 takes the x axis value (cosine component), and Mon-2 takes the y axis value (sine component) to represent a month. For example, March is represented as ([x, y] = [cos (-π/6), sin(-π/6)]), thereby Mon-1 = cos(-π/6) and Mon-2 = sin(-π/6).

The time variable (Time) indicates the temporal continuity (Figure S2B), defined as

Time = Year + (Month–1)/12.

For example, July 2015 was described as 2015 + (7-1)/12 = 2015.5. The total number of data pairs for the response and explanatory variables was 2520.

#### Hyperparameters tuning and model performance assessment

A 10-fold cross-validation method was used to optimize the hyperparameters and evaluate the accuracy of the model. The 10-fold cross-validation method divides the total data into ten sets, nine of which are training data, and the remaining one is test data. The function "stratified" from the "splitstackshape" package^38^ was used. In this case, the hyperparameters mtry (1–8) and ntree (100–15000) were tuned. The prediction accuracies on the test data are AUC (area under the ROC curve), CCI (correctly classified instances), MSE (mean squared error), NSE (Nash-Sutcliffe efficiency). To calculate the AUC, the function "roc" of the package "pROC" was used. The CCI indicates the accuracy of the model and is calculated using Equation 1 with a presence/absence threshold of 0.5.

(Equation 1) $$CCI=\frac{TP+TN}{TP+FP+FN+TN}$$

where TP, TN, FP, and FN are the true positive, true negative, false positive, and false negative, respectively. MSE is the mean squared error, which represents the difference between the observed values (Y_obs_) and the output values of the model (Y_sim_) and is calculated using Equation 2.

(Equation 2) $$MSE=\frac{1}{n}\sum_{i=1}^{n}{\left(Y_{\text{obs},i}-Y_{\text{sim},i}\right)}^{2}$$

NSE is a performance measure that considers the ratio of the model error and the variance in the observation data and is calculated using Equation 3:

(Equation 3) $$NSE=1-\frac{\sum_{i=1}^{n}{\left(Y_{\text{obs},i}-Y_{\text{sim},i}\right)}^{2}}{\sum_{i=1}^{n}{\left(Y_{\text{obs},i}-{\bar{Y}}_{\text{obs}}\right)}^{2}}$$

where *___Y*_obs_ denotes the mean observed value. COR is the correlation coefficient, which was calculated using the function "cor" in the package "stats." For the conventional species distribution modeling method, the models were first constructed on the dataset of the respective month(s), and the prediction results of each model were merged prior to the model performance assessment.

#### Post-hoc analysis for model interpretation

##### Variable importance

Variable importance indicates the relative contribution of each variable to the prediction accuracy of the model and allows us to understand the environmental factors that control species distributions. Predictions are performed on the post-hoc test data with shuffled values of the variable whose contribution is to be measured and the post-hoc test data without shuffling; the difference in their prediction accuracy indicates the contribution of the variable to the model prediction. This permutation-based evaluation was performed 100 times for all the variables, and the importance of each variable was compared. In the conventional species distribution modeling method, the importance of a variable at a specific period was assessed by applying the model built using the training data to the post-hoc test data (Figure 1). For the proposed method, the importance of a variable at a specific period was computed by applying the model built using the entire training dataset to post-hoc test data in a specific period (Figure 1). In this case, the function "model_parts" from the "DALEX" package^39^ was used to evaluate the variable importance.

##### Partial dependence plots

Partial dependence plots visualize the relationship between the explanatory and response variables. In a post-hoc test dataset (a pair of response and explanatory variables), the values of all variables except the target explanatory variable were fixed, and only the value of the target variable was changed to compute the model outputs along the range of the target variable. Lines are generated as many times as the size of the post-hoc test datasets, of which the mean line over these curves is the partial dependence plot for the given datasets. For the conventional method, partial dependence plots for a target period were generated using the same data as those used in model training. The proposed method uses post-hoc test data for a specific period to derive partial dependence plots.

Partial dependence plots can also be generated for temporal variables, and the procedure for their calculation is essentially the same as that for physical habitat variables. As the month variables (i.e., Mon-1 and Mon-2) were used to represent monthly cycles (Figure S2A), partial dependence plots for the month were computed using the two variables jointly and plotted along the circumference of the circle. Because the time variable (Time) is univariate and non-periodic, the partial dependence plot can be calculated independently. In addition to each month and time variable, a partial dependence plot considering both month and time simultaneously was derived to visualize the effect of all temporal information simultaneously. A partial dependence plot of the temporal information was computed using the three temporal variables jointly (Mon-1, Mon-2, and Time) and plotted along each point of the spiral diagram (Figure S2B).

The function "partial" of the "pdp" package^40^ was used to create partial dependence plot for the physical habitat variables. For the temporal variables (Mon-1, Mon-2, and Time), the above function does not work for a combination of multiple variables; thus, we coded to derive a partial dependence plot following the same procedures as the abovementioned package.

## References

[1] Tickner D., Opperman J.J., Abell R., Acreman M., Arthington A.H., Bunn S.E., Cooke S.J., Dalton J., Darwall W., Edwards G. (2020). Bending the Curve of Global Freshwater Biodiversity Loss: an Emergency Recovery Plan. Bioscience.

[2] Dudgeon D., Arthington A.H., Gessner M.O., Kawabata Z.I., Knowler D.J., Lévêque C., Naiman R.J., Prieur-Richard A.H., Soto D., Stiassny M.L.J., Sullivan C.A. (2006). Freshwater Biodiversity: Importance, Threats, Status and Conservation Challenges. Biol. Rev. Camb. Philos. Soc..

[3] Reid A.J., Carlson A.K., Creed I.F., Eliason E.J., Gell P.A., Johnson P.T.J., Kidd K.A., MacCormack T.J., Olden J.D., Ormerod S.J. (2019). Emerging Threats and Persistent Conservation Challenges for Freshwater Biodiversity. Biol. Rev. Camb. Philos. Soc..

[4] Tonkin J.D., Altermatt F., Finn D.S., Heino J., Olden J.D., Pauls S.U., Lytle D.A. (2018). The Role of Dispersal in River Network Metacommunities: Patterns, Processes, and Pathways. Freshw. Biol..

[5] Belletti B., Garcia de Leaniz C., Jones J., Bizzi S., Börger L., Segura G., Castelletti A., van de Bund W., Aarestrup K., Barry J. (2020). More Than One Million Barriers Fragment Europe’s Rivers. Nature.

[6] Grill G., Lehner B., Thieme M., Geenen B., Tickner D., Antonelli F., Babu S., Borrelli P., Cheng L., Crochetiere H. (2019). Mapping the World’s Free-Flowing Rivers. Nature.

[7] Lennox R.J., Paukert C.P., Aarestrup K., Auger-Méthé M., Baumgartner L., Birnie-Gauvin K., Bøe K., Brink K., Brownscombe J.W., Chen Y. (2019). One Hundred Pressing Questions on the Future of Global Fish Migration Science, Conservation, and Policy. Front. Ecol. Evol..

[8] Guisan A., Thuiller W. (2005). Predicting Species Distribution: Offering More Than Simple Habitat Models. Ecol. Lett..

[9] Zimmermann N.E., Edwards T.C., Graham C.H., Pearman P.B., Svenning J.C. (2010). New Trends in Species Distribution Modelling. Ecography.

[10] Araújo M.B., Guisan A. (2006). Five (or So) Challenges for Species Distribution Modelling. J. Biogeogr..

[11] Martínez-Minaya J., Cameletti M., Conesa D., Pennino M.G. (2018). Species Distribution Modeling: a Statistical Review With Focus in Spatio-temporal Issues. Stoch. Environ. Res. Risk Assess..

[12] Elith J., Leathwick J.R. (2009). Species Distribution Models: Ecological Explanation and Prediction Across Space and Time. Annu. Rev. Ecol. Evol. Syst..

[13] Chuine I. (2010). Why Does Phenology Drive Species Distribution?. Philos. Trans. R. Soc. Lond. B Biol. Sci..

[14] Ponti R., Sannolo M. (2022). The Importance of Including Phenology When Modelling Species Ecological Niche. Ecography.

[15] Ryo M., Yoshimura C., Iwasaki Y. (2018). Importance of antecedent environmental conditions in modeling species distributions. Ecography.

[16] Huang J., Frimpong E.A., Orth D.J. (2016). Temporal Transferability of Stream Fish Distribution Models: Can Uncalibrated SDMs Predict Distribution Shifts Over Time?. Divers. Distrib..

[17] McNyset K.M. (2005). Use of Ecological Niche Modelling to Predict Distributions of Freshwater Fish Species in Kansas. Ecol. Freshw. Fish.

[18] Gschweng M., Kalko E.K.V., Berthold P., Fiedler W., Fahr J. (2012). Multi-temporal Distribution Modelling With Satellite Tracking Data: Predicting Responses of a Long-Distance Migrant to Changing Environmental Conditions. J. Appl. Ecol..

[19] Baltensperger A.P., Joly K. (2019). Using Seasonal Landscape Models to Predict Space Use and Migratory Patterns of an Arctic Ungulate. Mov. Ecol..

[20] Lucas T.C.D. (2020). A Translucent Box: Interpretable Machine Learning in Ecology. Ecol. Monogr..

[21] Breiman L. (2001). Random Forests. Mach. Learn..

[22] Ryo M., Harvey E., Robinson C.T., Altermatt F. (2018). Nonlinear Higher Order Abiotic Interactions Explain Riverine Biodiversity. J. Biogeogr..

[23] Fukuda S., De Baets B., Waegeman W., Verwaeren J., Mouton A.M. (2013). Habitat Prediction and Knowledge Extraction for Spawning European Grayling (*Thymallus thymallus* L.) Using a Broad Range of Species Distribution Models. Environ. Model. Softw..

[24] Williams H.M., Willemoes M., Thorup K. (2017). A temporally explicit species distribution model for a long distance avian migrant, the common cuckoo. J. Avian Biol..

[25] Katano O. (1992). Spawning Tactics of Paired Males of the Dark Chub, *Zacco temmincki*, Reflect Potential Fitness Costs of Satellites. Environ. Biol. Fishes.

[26] Carter M.G., Copp G.H., Szomlai V. (2004). Seasonal abundance and microhabitat use of bullhead Cottus gobio and accompanying fish species in the River Avon (Hampshire), and implications for conservation. Aquat. Conserv..

[27] Nagayama S., Negishi J., Kume M., Sagawa S., Tsukahara K., Miwa Y., Kayaba Y. (2012). Habitat Use by Fish According to Seasons and Life Stages in Small Perennial Agricultural Canals. Ecol. Civ. Eng..

[28] Hernandez P.A., Graham C.H., Master L.L., Albert D.L. (2006). The effect of sample size and species characteristics on performance of different species distribution modeling methods. Ecography.

[29] Thibaud E., Petitpierre B., Broennimann O., Davison A.C., Guisan A. (2014). Measuring the relative effect of factors affecting species distribution model predictions. Methods Ecol. Evol..

[30] Wisz M.S., Hijmans R.J., Li J., Peterson A.T., Graham C.H., Guisan A., NCEAS Predicting Species Distributions Working Group (2008). Effects of sample size on the performance of species distribution models. Divers. Distrib..

[31] Randin C.F., Dirnböck T., Dullinger S., Zimmermann N.E., Zappa M., Guisan A. (2006). Are Niche-Based Species Distribution Models Transferable in Space?. J. Biogeogr..

[32] Champion C., Hobday A.J., Pecl G.T., Tracey S.R. (2020). Oceanographic Habitat Suitability Is Positively Correlated With the Body Condition of a Coastal-Pelagic Fish. Fish. Oceanogr..

[33] Katano O., Baba Y., Ohara H., Kawamura K., Sato M., Kumagaya M., Takeuchi M., Ito S., Togashi S., Inoue N. (2014). Enlarged Distribution of *Nipponocypris temminckii* as a Domestic Alien Fish. Jpn. J. Ichthyol..

[34] Ohira M., Nishida K., Mitsuo Y., Tsunoda H., Senga Y. (2008). Longitudinal Distribution of Fishes and Environmental Conditions in a Small Basin of Low-Altitude. J Environ Inf Sci.

[35] Cutler D.R., Edwards T.C., Beard K.H., Cutler A., Hess K.T., Gibson J., Lawler J.J. (2007). Random Forests for Classification in Ecology. Ecology.

[36] Liaw A., Wiener M. (2002). Classification and Regression by randomForest. R. News.

[37] R Core Team (2020). https://www.R-project.org/.

[38] Mahto A. (2019). Splitstackshape: stack and reshape datasets after splitting concatenated values. https://cran.r-project.org/web/packages/splitstackshape/splitstackshape.pdf.

[39] Biecek P. (2018). DALEX: Explainers for Complex Predictive Models in R. J. Mach. Learn. Res..

[40] Greenwell B.M. (2017). pdp: an R Package for Constructing Partial Dependence Plots. R J..

