# Supplementary material for: Temporal variables improve a spatiotemporal species distribution model for the non-native freshwater fish Candidia temminckii
Source: iScience. 2024 Mar 6;27(4):109445. doi: 10.1016/j.isci.2024.109445 (PMC10972797; doi:10.1016/j.isci.2024.109445)
Supplement: Document S1. Figures S1–S8 and Table S1 [file mmc1.pdf]

iScience, Volume *27*

Supplemental information

Temporal variables improve a spatiotemporal species distribution model for the non-native freshwater fish *Candidia temminckii*

Taichi Jibiki and Shinji Fukuda

Supplemental information

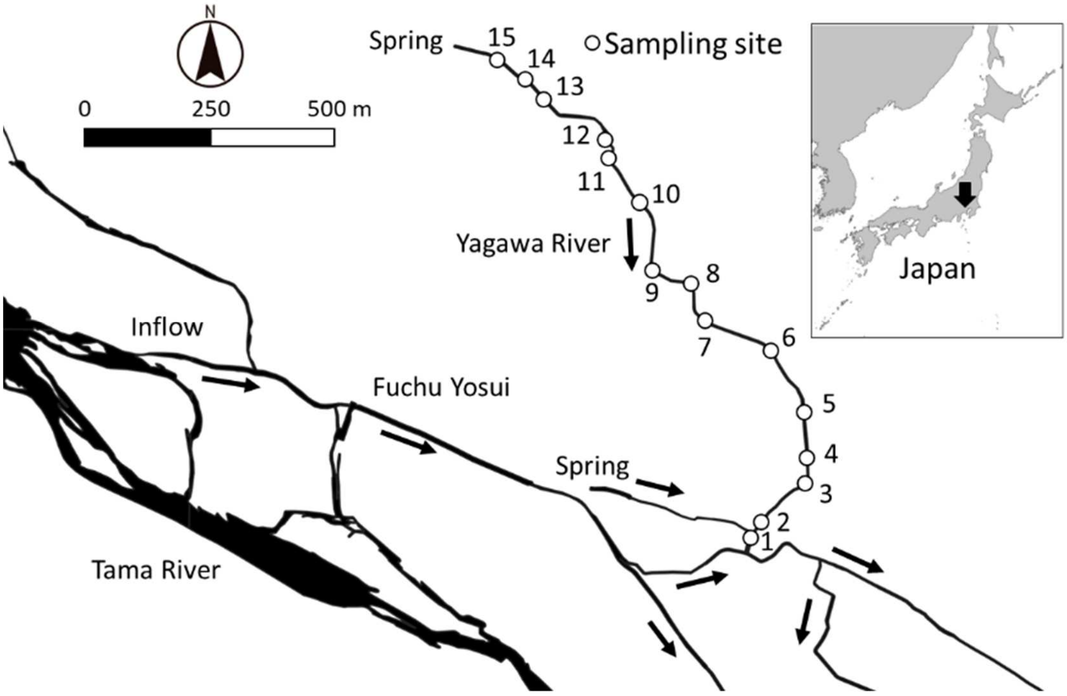

Figure S1 Location of study area and sampling sites. The target site is the Yagawa River, which flows through Kunitachi, Tokyo, Japan. The Yagawa River is a spring-fed river, approximately 1.4 km in length, and its downstream end is connected to the Fuchu Yosui irrigation system.

Table S1 Data partitioning methods for conventional and proposed methods. The conventional methods split an entire dataset based on the time window of interest. The proposed method builds one model with the entire dataset and interprets the pattern of each period using post-hoc analyses.

	Months											
Conventional-12	12	1	2	3	4	5	6	7	8	9	10	11
Conventional-6	12	1	2	3	4	5	6	7	8	9	10	11
Conventional-3	12	1	2	3	4	5	6	7	8	9	10	11
Conventional-1	12	1	2	3	4	5	6	7	8	9	10	11
Proposed	12	1	2	3	4	5	6	7	8	9	10	11

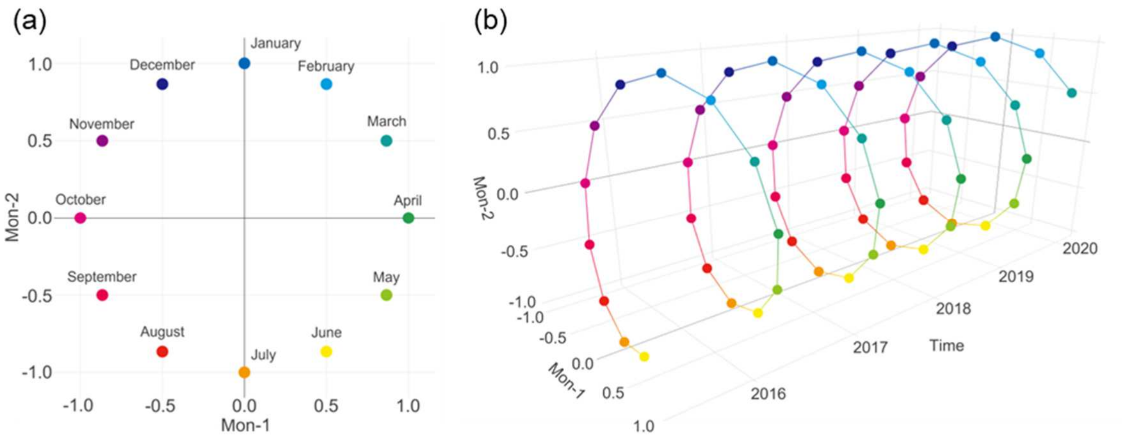

Figure S2 Conceptual diagrams showing the temporal variables: (a) month and (b) time. The monthly variable describes the periodic similarities. To represent a cycle, two variables (Mon-1 (x-axis) and Mon-2 (y-axis)) are used and positioned equally spaced clockwise from the starting point ([x, y] = [0, 1]) in a circle with a radius of 1. Mon-1 takes the x-axis value (cosine component) and Mon-2 takes the y-axis value (sine component) to represent a month. The time variable (Time) indicates temporal continuity.

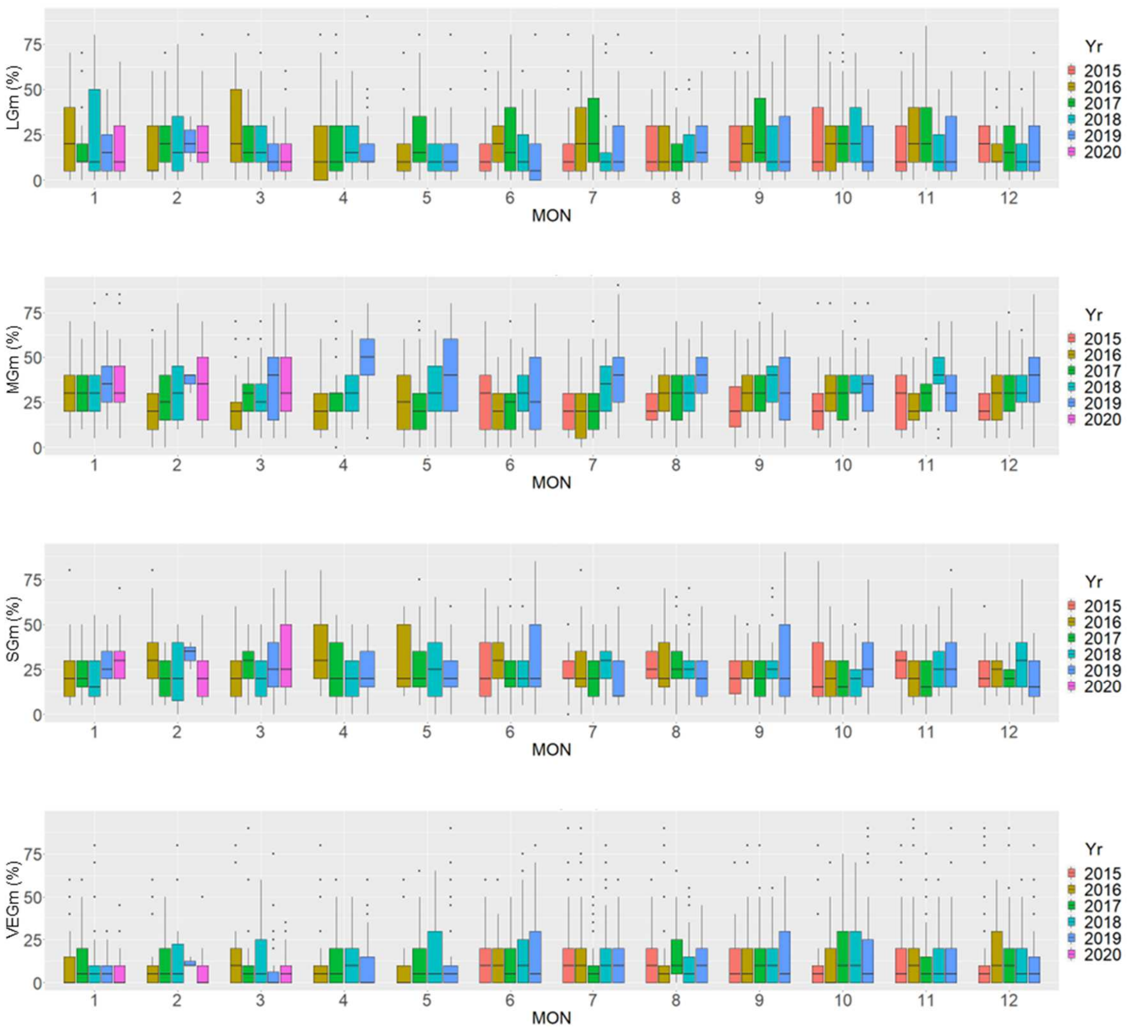

Figure S3 Temporal dynamics of physical habitat conditions related to substrates and vegetation: percent coverage of large-sized gravel (LGm), medium-sized gravel (MGm), small-sized gravel (SGm), and vegetation (VGm). The horizontal axis of the figure indicates the month (MON) in which the survey was conducted.

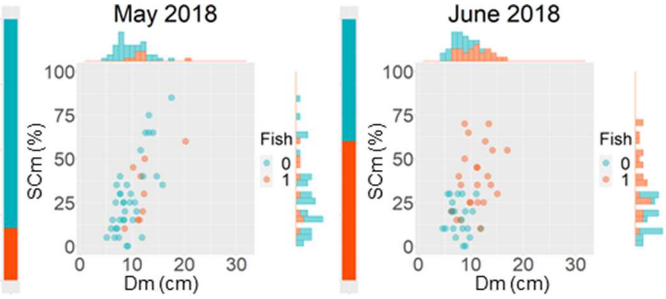

Figure S4 Comparison of the distribution of physical habitat variables (water depth (Dm) and percent coverage of sand and clay (SCm)). The red/green bar represents the ratio of the presence and absence of fish at the survey sites in consecutive months of the year (May 2018 and June 2018).

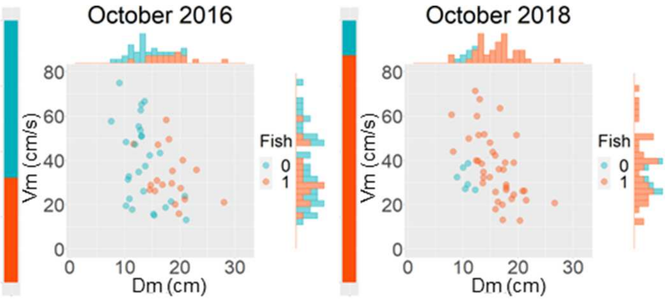

Figure S5 Comparison of the distribution of physical habitat variables (Dm and Vm). The red/green bar represents the ratio of the presence and absence of fish at the survey sites in the same months, but in different years (October 2016 and October 2018).

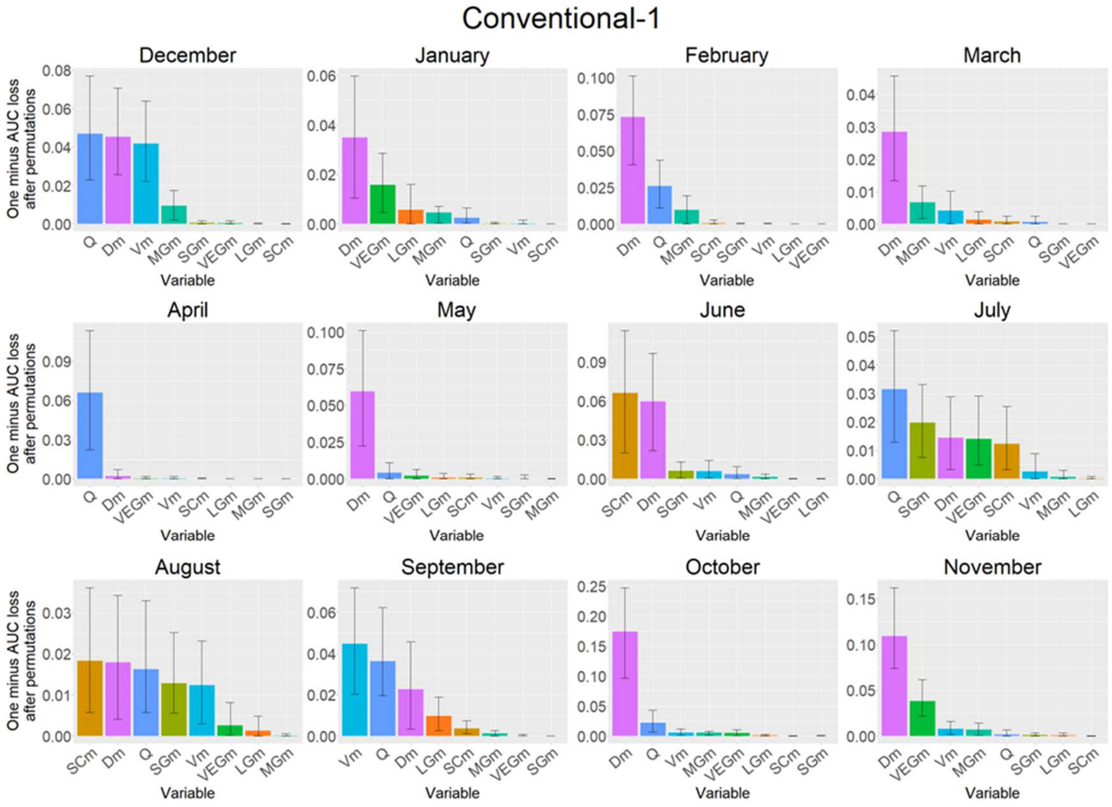

Figure S6 Variable importance computed by the conventional method (case: Conventional-1). The y-axis shows the importance of each variable in the model prediction. The values of the bars represent the mean variable importance calculated using 100 permutations and the error bars indicate the maximum and minimum values of importance. The explanatory variables are the mean depth (Dm), mean velocity (Vm), flow discharge (Q), percent coverage of aquatic vegetation (VEGm), large-sized gravel (LGm), medium-sized gravel (MGm), small-sized gravel (SGm), and silt and sand (SCm).

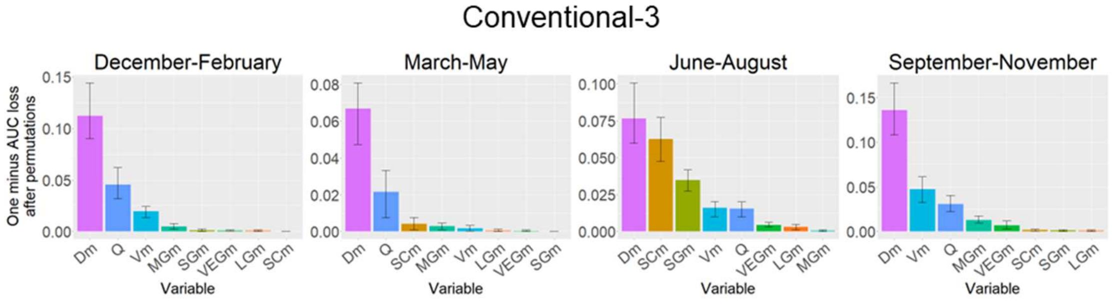

Figure S7 Variable importance computed by the conventional method (case: Conventional-3). The y-axis shows the importance of each variable in the model prediction. The values of the bars represent the mean variable importance calculated using 100 permutations and the error bars indicate the maximum and minimum values of importance. The explanatory variables are the mean depth (Dm), mean velocity (Vm), flow discharge (Q), percent coverage of aquatic vegetation (VEGm), large-sized gravel (LGm), medium-sized gravel (MGm), small-sized gravel (SGm), and silt and sand (SCm).

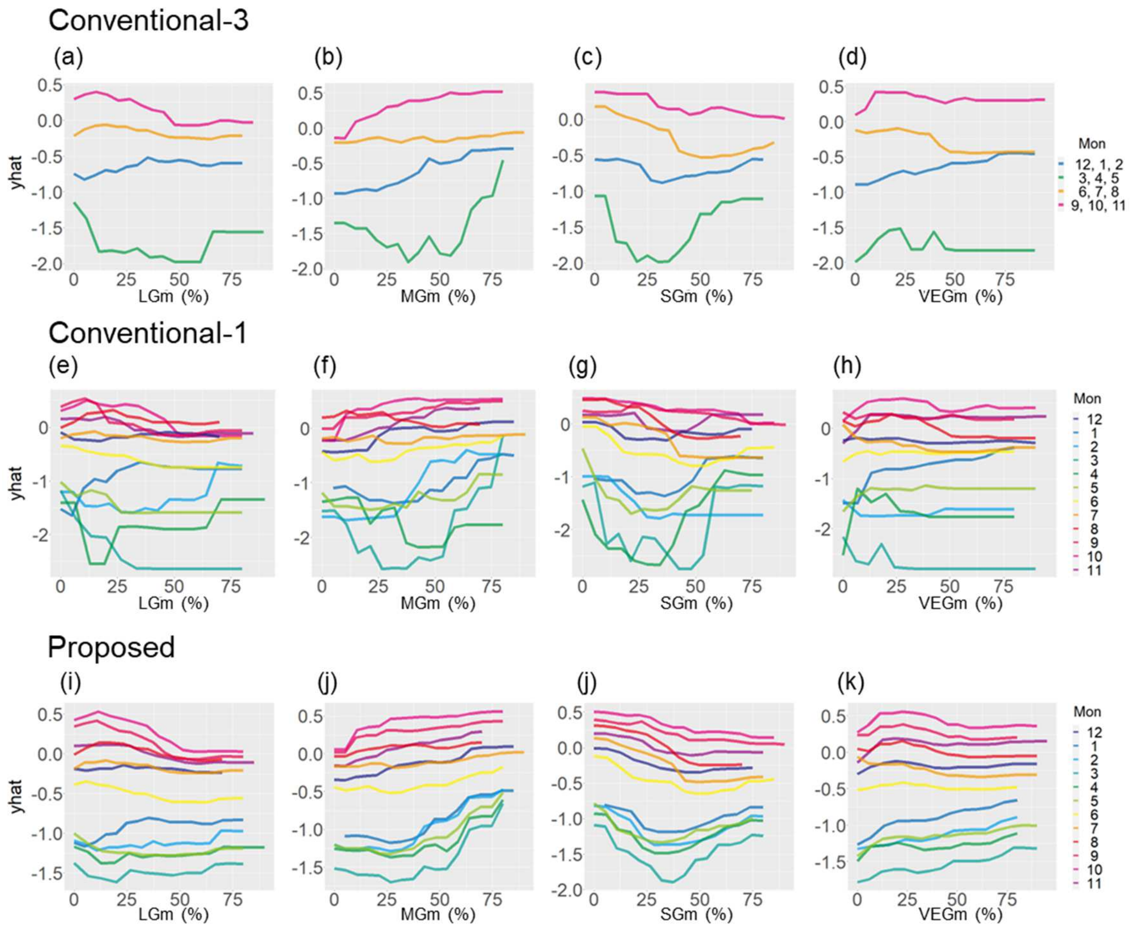

Figure S8 Partial dependence plot for physical habitat variables by conventional (case: Conventional-3 and Conventional-1) and proposed methods: percent coverage of large-sized gravel (LGm), medium-sized gravel (MGm), small-sized gravel (SGm), and vegetation (VGm). Partial dependence plots show the relationship between model predictions and changes in the values of the explanatory variables. The y-axis shows the trend of change in the model's predicted results.
